# The physical activity parenting practices (PAPP) item Bank: a psychometrically validated tool for improving the measurement of physical activity parenting practices of parents of 5–12-year-old children

**DOI:** 10.1186/s12966-020-01036-0

**Published:** 2020-11-04

**Authors:** Louise C. Mâsse, Teresia M. O’Connor, Yingyi Lin, Nicole S. Carbert, Sheryl O. Hughes, Tom Baranowski, Mark R. Beauchamp

**Affiliations:** 1 BC Children’s Hospital Research Institute, School of Population and Public Health University of British Columbia, F508-4490 Oak Street, Vancouver, BC Canada; 2 USDA/ARS Children’s Nutrition Research Center, Baylor College of Medicine, 1100 Bates St, Houston, TX 77030 USA; 3 School of Kinesiology, University of British Columbia, 210-6081 University Blvd, Vancouver, BC V6T 1Z1 Canada

**Keywords:** Physical activity parenting practices, Validity, Reliability, Measurement, Questionnaire, Children, Parents

## Abstract

**Background:**

Many tools have been developed to measure physical activity parenting practices (PAPP). Currently, there is little standardization on how PAPP constructs are operationalized for 5–12 year-old children. Given this lack of consistency our team have started the process of standardizing the measurement of PAPP by developing an item bank which was conceptually informed by 24 experts from 6 countries.

**Purpose:**

The purpose of this paper is to present the psychometric properties of the PAPP item bank using the expert-informed PAPP conceptual framework.

**Methods:**

A sample (*N* = 626) of Canadian parents completed the PAPP item bank (100 items measuring 12 constructs). Confirmatory Factor Analyses (CFA), confirmatory bi-factor item analyses, and Item Response Modeling (IRM) were used to assess the structural validity of scores derived from the PAPP item bank. Differential Item Functioning (DIF) and Differential Response Functioning (DRF) were used to determine whether the PAPP items are invariant by parent sex, ethnicity of parent, and household income. Finally, Computerized Adaptive Testing (CAT) simulations were used to determine the efficiency of the item bank – this involved ascertaining whether each construct can be assessed with fewer items.

**Results:**

The PAPP expert-informed conceptual framework was mainly supported by the CFA analyses. Notable changes included: a) collapsing smaller constructs into one general construct (modeling, co-participation, and monitoring constructs were collapsed into a construct assessing nondirective support); or b) splitting a construct into two smaller constructs (restrict for safety reason construct was split into indoor physical activity restriction and allowance for unsupervised outside physical activity). While the CFA analyses supported the structural validity of 11 constructs, the bi-factor item analyses and IRM analyses supported collapsing correlated constructs into more general constructs. These analyses further reduced the number of constructs measured by the PAPP item bank to nine constructs (65 items – reliability ranging from .79 to .94). As seven of the PAPP constructs had reliability greater than .80, CAT simulations further reduced the number of items to 31 items.

**Conclusion:**

Overall, the PAPP item bank has excellent psychometric properties and provides an efficient way to assess PAPP.

**Supplementary information:**

The online version contains supplementary material available at 10.1186/s12966-020-01036-0.

## Introduction

The benefits of physical activity (PA) have been well established [1]. In children 6–17 years of age, participation in PA is associated with improved cardiorespiratory and muscular fitness, bone health, cognitive functioning (e.g., executive function, memory and attention), and academic performance [1]. Children who participate in PA also have fewer cardiovascular risk factors and psychological impairments [1]. To gain the benefits associated with PA participation, current global recommendations suggest that children 5 to 17 years of age accumulate at least 60 min of moderate to vigorous PA daily [2]. However, in North America about a third of children 6–17 years of age do not meet current PA guidelines [3–5]. Furthermore, trends over time show that levels of PA in children have not historically changed and that participation in PA decreases in adolescence by as much as 60 to 75% from childhood [3, 6–8].

There are numerous social and environmental factors that can influence children’s participation in PA. In this study, we focus on the role of parents as a growing body of research highlights the role of parenting practices on children’s participation in PA [9, 10]. Parenting practices refer to the specific goal directed strategies that parents use to influence their child’s behaviours, including their PA [11]. A recent review concluded that parental role modeling of PA and provision of logistic support for PA were more consistently associated with children’s level of PA across studies than other PA parenting practices (e.g., monitoring and encouraging PA) [10]. The evidence for role modeling and parental logistic support influencing children’s PA was stronger from qualitative studies (87.5 and 62.5%, respectively) than quantitative studies (23.3 and 53.3%, respectively) [10]. Other aspects of PA parenting practices, including PA monitoring and encouragement, are associated with children’s level of PA; however, the evidence remains unclear as few studies have focused on these constructs and in some cases the results are contradictory [10, 12]. For example, Arrerondo et al. [13] found a positive association between PA monitoring and child PA in a sample of young children whereas Bradley et al.’s [14] study found that 9–15 yr. old boys with less parental monitoring but more encouragement had higher levels of PA. Comparing findings across studies or trying to assess the true impact of parenting practices on children’s PA behaviours remains challenging as there is considerable variation and little consistency in how PA parenting practices are operationalized [9, 12, 15].

A recent review identified more than 74 tools for measuring PA parenting practices of 5–12 year-old children [16]. These measures varied in their conceptual influences depending on the field in which they were developed (e.g., physical activity or psychology) and whether they were influenced by any theoretical perspectives [9]. There is a need to bring more rigour to PA parenting practice measures by integrating knowledge from the parenting literature in the operationalization of PA parenting practices constructs [9, 17]. Our team initiated a process to help standardize the measurement of PA parenting practices of 5–12 year-old children [16]. Specifically, concept mapping methodology was utilized to engage 24 experts from 6 countries in reconceptualising the constructs of PA parenting practices with the parenting literature as the underlying framework. This process identified 12 constructs covering three main domains of parenting namely structure, neglect/control, and autonomy promotion [16]. Following this process, an item bank of 100 PA parenting practice items covering 12 PA parenting concepts was developed.

The ultimate aim of this paper is to develop a repository of calibrated items to standardize the measurements of PA parenting practices among school age children (5–12 year-old children). Using the NIH PROMIS initiative [18] procedures, the specific aims of this study were to: a) Assess the structural validity of the PA Parenting Practices (PAPP) item bank. Using Confirmatory Factory Analyses (CFA), we tested whether the PAPP item bank replicated the underlying factorial structure of the published PA conceptual framework which includes 3 domains and 12 constructs to measure PAPP [16]. As many of the constructs within each parenting domain were expected to be highly correlated, we used advanced psychometric methodologies (confirmatory bi-factor item analyses with Item Response Modeling – IRM) to explore whether the hypothesized conceptual framework could be simplified to measure more general constructs; b) Determine whether the psychometric properties of the constructs or general constructs are invariant by parent sex, ethnicity of parent, and household income; and c) Determine the efficiency of the item bank – which involves assessing whether each construct or general construct can be assessed with fewer items.

This paper used both classical (CFA) and more advanced psychometric methods (confirmatory bi-factor item analyses) to develop more efficient ways of assessing PAPP – a methodological approach that has not been used in this field. Measuring the breadth of PAPP with fewer constructs and items is necessary to ensure uptake of these measures in practice.

## Methods

### Participants

Canadian parents of 5–12-year-old children were recruited from an internet research polling firm (InSight West, Canada) to complete the (Physical Activity Parenting Practice) PAPP questionnaire. Data were collected from November 2016 until January 2017. All participants previously consented to be part of the InSight West web-based panel. Participants were required to be a parent or guardian of a 5–12-year-old child. Participants were excluded if their child had a disability that limited their participation in any PA. A quota sampling approach was used to ensure representation by parent’s sex, ethnicity (Caucasian, East/Southeast Asian, South/West Asian and others), and income (using the 2015 median income of double and single income earners accordingly). Given the length of the PAPP questionnaire, parents completed the questionnaire in two waves about 2 weeks apart. Of the 945 panel members who completed the screener, 144 did not meet eligibility criteria, 158 dropped out, 16 opened the wave 1 questionnaire but did not respond to the parenting questions, and 626 completed the wave 1 questions (response rate = 66%). Of these, 479 completed the wave 2 questions (response rate 51%). Demographic characteristics of the sample are shown in Table 1. Ethics approval for conducting this study was obtained from the first author’s Research Ethics Board before study commencement.

**Table 1:** Demographic characteristics of participants (*N* = 626)

	% or Mean (SD)
Parent Sex (*N* = 626) Female	50.3%
Parent Age (*N* = 626) Years	42.1 (7.68)
Marital status (*N* = 626)	
Married or common law	86.7%
Separated or divorced	7.3%
Never married	5.9%
Ethnicity (*N* = 626)	
Caucasian	50.8%
Asian	22.5%
South Asian	14.9%
Other	11.8%
Education (*N* = 458)	
High school or less	9.6%
Certificate non-university or some college or university	35.2%
Bachelor’s degree	32.5%
Postgraduate degree	17.7%
Professional degree	5.0%
Income Cdn (*N* = 626)	
Less than $50,000	17.3%
$50,000 – 69,999	16.5%
$70,000 – 79,999	10.2%
$80,000–99,999	18.4%
$100,000 – 124,999	13.7%
$125,000 – 149,999	10.4%
$15,000 or higher	13.6%
Self-Reported Body Mass Index (*N* = 430)	
Underweight	1.9%
Normal	40.7%
Overweight	33.7%
Obese	23.7%
Child Sex (N = 626) Female	48.4%
Child age (*N* = 626)	
5–8 year old	50.0%
9–12 year old	50.0%

### Development of physical activity parenting practices (PAPP) item bank

The PAPP conceptual framework, [16] developed from expert input by our team, guided the development of the PAPP item bank. Briefly, the PAPP conceptual framework identified 12 constructs that regrouped onto three main domains of parenting practices which have been widely used in developmental psychology [11, 19, 20] and recently adopted in the field of PA [9]. Specifically, the PAPP conceptual framework include 12 constructs that regrouped into the following three domains of PAPP: a) the neglect/control; b) the autonomy promotion, and c) the structure (for a full description see ref. [16]. Briefly, the neglect/control domain of PA parenting practices includes two constructs that measure permissive, coercive, and pressuring parenting practices. By regrouping these two constructs, two parenting practice concepts believed to be ineffective are brought together. The coercive and pressuring components includes criticizing, nagging, forcing, and punishing while the permissiveness is defined as not providing any parental guidance and allowing the child to make decisions about their PA. The autonomy promotion domain regroups four constructs (encouragement, guided choice, parental involvement in child PA, and praising/rewarding child) that assess how responsive parents are to their child’s PA needs. Encouragement includes verbal strategies that parents use to motivate child PA such as positive verbal reinforcement, reasoning, and highlighting behaviours of role models. Guided choice includes strategies that parents use to support their child’s independence and the strategies they use to involve their child into PA decisions such as choosing PA options and negotiating with the child. The parental involvement in child PA construct measures the extent to which parents are involved in their child’s PA/sport (e.g., talk about their sports, watch them participate, teach them skills to improve their skills, and volunteer in their activities). The praise/reward construct measures the strategies parents use to reinforce participation without coercing participation such as providing a small token of appreciation. Finally, the structure domain of parenting includes the strategies that parents employ to ensure their child participates in PA and regroups six constructs. These include a) co-participation - the extent to which parents are physically active with their child; b) expectations - whether parents have clear expectations for PA participation; c) facilitation - the tangible ways in which parents support PA participation such as enrolling, transporting to activities, taking children places to be active, and providing the financial support to be active); d) modeling - the extent to which parents are engaged in PA and model an active lifestyle; e) monitoring - involves tracking or being aware of their child’s level of PA; and f) restrictions for PA related to safety or academic concerns - includes the reasons why parents may limit involvement in PA.

Following the development of the PAPP conceptual framework, our team populated the item bank to cover the three domains and 12 constructs by selecting items from three different sources: 1) items from previous instruments (74 instruments and 608 items) which we identified from a review of the literature [16]; 2) items that had been developed to match the strategies parents reported using to encourage or discourage their child to be physically active – where 135 parents were asked 5-open ended questions and the data were qualitatively analyzed [21]; and 3) new items developed for this purpose when few items were available from the first two sources or the content did not represent the breath of the construct assessed. As many items were taken from various sources, all items and response formats were standardized. LCM and TMO took the lead in developing and standardizing the items and ensuring that the breadth of the content aligned with the operational definitions of each construct [16]. The content of the item bank (100 items) was iteratively reviewed by the larger team of investigators (MB, SOH, & TB). The items were then cognitively tested using both a think-aloud protocol and probing protocols with a total of 10 Canadian parents [22]. As the PAPP item bank included 100 items, the think-aloud protocol was primarily used to review newly developed items. This involved asking the parents to read each item out aloud and: a) verbalize their understanding of the items; b) state the process they used to retrieve a relevant response to the item, and c) articulate how they went about selecting a given response. As part of this process, parents were asked whether the response format was appropriate to determine whether certain items were best asked with a generic response format (using a 5-point response format ranging from never to very often) or a more specific response format (using a response format that quantified the number of times they used a given parenting practice ranging from never, 1–2 times per month, 3–4 times per month, etc. …). For the remaining items, parents were asked to complete 1 page of the survey at a time and asked to indicate whether some items were unclear, difficult to answer, or whether they could not answer a given item. Essentially, parents were asked to go through the think-aloud protocol on their own but to discuss with the interviewer any issues they encountered. As part of this process, the interviewer asked probing questions at the end of each page (e.g., whether they understood specific questions, struggled with mapping their response to the format used). This process continued until the full questionnaire was completed. This process was used to develop the items and prepare them for the psychometric evaluation. For the analyses, only the items that pertained to PA were analyzed (96 items) and the 4 items that pertained to screen time were dropped from the analyses.

### Data collection

The participants completed the PAPP items in two waves as its length made it impossible to complete it in one administration, with all administrations completed online. At wave 1, the 626 web-based panel members completed the demographic questions and were administered 76 of the 96 PAPP items. The amount of missing data for these 76 PAPP items ranged between 5% to less than 1%. One to 2 weeks after completing the wave 1 questionnaire, the participants completed the remaining 20 PAPP items. A total of 479 (77%) participants completed the wave 2 questionnaire and among those who completed the wave 2 questionnaire the amount of missing data for the 20 PAPP items ranged between 4% to less than 1%, except for one item (19% missing data). To determine whether the pattern of responses differed between those who completed only the wave 1 PAPP items (*N* = 626–479 = 147) versus those who completed both waves of data collection (*N* = 479), chi-square tests and t-tests were conducted. The 76 PAPP items administered at wave 1 served to assess whether the pattern of responses were similar between wave 1 respondents and respondents of both waves. For the 76 items, the pattern of responses was not significantly different between responders (*p* < .01). In addition, the demographic characteristics (parental sex, age, ethnicity, education, and income as well as child sex and age) of the wave 1 responders versus the respondents of both waves were not significantly different (*p* < .01). This suggest that the data are likely missing at random. The results section provides the list of items that were administered and completed only by the wave 2 participants.

### Structural validity of the PAPP item bank (aim 1)

#### Confirmatory factor analyses and confirmatory bi-factor item analyses

Initial Confirmatory Factor Analysis (CFA) assessed whether the 12 PAPP constructs were structurally supported. As the CFAs were followed by confirmatory bi-factor item analyses, CFAs were conducted for each domain. This was done as we assumed that correlated constructs within each domain could be simplified into more general construct(s) that would be conceptually meaningful. Analyses tested the following hypotheses of whether: a) the neglect/control domain included two constructs that measured permissive and pressuring parenting practices; b) the autonomy promotion domain included four constructs that measured encouragement, guided choice, parental involvement in child physical activities, and praising/rewarding child for being active; and c) the structure domain included six constructs that measured co-participation, expectations, facilitation, modeling, monitoring, and restrictions for PA related to safety [16]. Note that the original conceptual framework for restrictions also included restrictions for academic concerns. Restriction for academic concerns was not operationalized as the questions overlapped with the facilitations questions about enrollment and so we opted to not assess the reasons for not enrolling in PA.

**Fig. 1:**
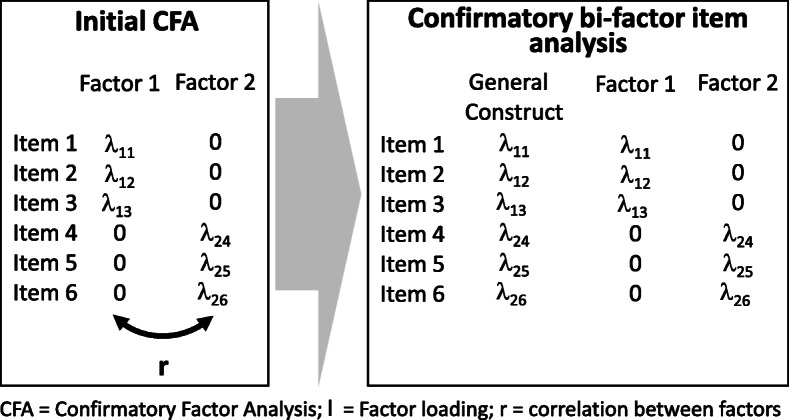
Analytical steps to regroup correlated constructs into general constructs

As many of the constructs within the PAPP domains were significantly correlated (r ≥ .70), a confirmatory bi-factor item analysis followed these initial CFAs to assess whether the constructs for a given domain of parenting practices can be collapsed into a simpler structure. Fig. 1 provides a schematic of these analyses. After a suitable bi-factor structure was identified, the analyses proceeded to select items that measured general constructs of PAPP but conformed to an “essentially unidimensional” structure which confirms that items measure a construct that is on the same continuum.

MPlus version 8 was used for the CFA and confirmatory bi-factor item analyses [23]. To deal with the ordinal nature of the data and missing data, the weighted least squares means and variance-adjusted (WLSMV) estimation was used with a full information procedure (approach used to deal with the missing data) [24]. As there are no agreed standards to assess model fit with CFA or the confirmatory bi-factor analyses, a number of indices were reviewed to assess overall model fit: Steiger’s Root Mean Square Error of Approximation (RMSEA), with an upper value of 0.08 indicative of a reasonable fit, although some suggest a cut-off of 0.10 might be indicative of reasonable fit with more complex models; the Comparative Fit Index (CFI) with values ≥0.95 suggestive of a good fit; and the weighted root mean square residual (WRMR) with a value closer to 1 indicative of a good fit [25–28]. As part of the confirmatory bi-factor item analyses, the I-ECV (Explained Common Variance for a Single Item) [29] index was computed to identify items that were strongly related to the general construct. The I-ECV was computed as a ratio of the squares of the item’s loading on the general construct divided by the sum of the squares of the general and the specific construct the item measured. Items that had an I-ECV ≥ .70 and/or a factor loading on the general construct approaching .50 were retained for the IRM analyses [29]. Items with lower I-ECV or factor loadings were retained only if it made conceptual sense but this strategy was seldom used.

#### Item response modeling (IRM) analyses

After initial candidate items were selected, unidimensional IRM models were fitted for each construct identified using the MIRT function in R statistical package [30]. A two-parameter graded response model was fitted to the data using the Expectation Maximization algorithm with missing data imputed. To determine whether local dependence among the items was sufficiently addressed, the residual correlations were evaluated. Any residual correlations greater than .25 were evaluated and items were further deleted to ensure that the analysis satisfied the IRM assumption of local dependence. Overall model fit was assessed with the M^2^ chi-square statistic and the RMSEA, CFI, and SRMR using the criteria above and a value between .05 to .08 for the SRMR as indicative of good fit [31]. These analyses served to reassess the unidimensionality of the constructs. Note that the fit indices were available when the construct had 8 or more items but local dependence could be assessed with fewer items. Most importantly, these analyses generated the item parameters (i.e., discrimination and difficulty parameters) and item information, which are needed to assess the efficiency of the item bank (aim 3) and identify the most informative items.

### Invariance properties of the item bank (aim 2)

To ensure the constructs measured by the PAPP item bank can be used to make valid group comparisons, differential item functioning (DIF) and differential response functioning (DRF) analyses were conducted using the MIRT R Software [32]. The aim of these analyses was to assess whether sub-groups of parents who have similar scores on a given construct had similar patterns of responses. If the scores were similar, it indicated that the scores resulted in similar interpretations across groups. Potentially invariant items were first assessed with the DIF analyses, followed by the DRF analyses. Invariance was tested only for constructs that had more than three items, as DRF cannot be assessed with fewer items. DRF tests for two types of response bias: 1) signed-DRF which tests whether bias is consistent across all scores in a given construct; and 2) unsigned-DRF which tests whether there is an interaction in the bias across scores on a given construct. Items retained from the structural validity analyses were assessed for DRF by parents’ sex, income, and ethnicity. Items identified to have significant DRF (*p* < .01) between groups were removed from the item bank.

### Efficiency of the PAPP item bank and informative items (Aim3)

While the primary purpose of our work was to develop an item bank of PAPP, shorter measures may be needed for some applications. Therefore, for any constructs that had a reliability >.80, the FIRESTAR software was used to help determine how to shorten the scale [33]. Briefly, FIRESTAR uses Computerized Adaptive Testing (CAT) simulation to assess how many items, and which items, should be retained to preserve a reliability for a given scale ≥ .80. The CAT simulations used the graded response model with the maximum posterior weighted information to select items. The maximum standard error for the estimate was set at .447 which corresponds to a reliability of .80. Input for these analyses were the item parameters estimated from the IRM analyses.

### Reliability of constructs

Cronbach alpha was used to assess the reliability of the responses following the CFAs. In addition, the IRM empirical reliability was also reported and this reliability is similar to Cronbach alpha except that it takes into account the ordinal nature of the responses (Likert type scales).

## Results

### Neglect/control domain of PAPP

#### Structural validity

As shown in Table 2, the hypothesized 2-factor structure for the neglect/control PAPP domain was supported by the initial CFA analyses. Given that the initial CFA found a high correlation between the permissive and pressure constructs (r = .85), it was not surprising that the results of the bi-factor item analysis found that all items loaded highly on the general dimension (coercive control), except for item 1 “Allow child to stay inside” (as the I-ECV for this item was less than .50 it was dropped) (see Tables 2 and 3). The IRM analyses further supported the unidimensionality of the 19 items as the model had an adequate fit and no local dependence was observed.

**Table 2:** Overview of the Confirmatory Factor Analyses (CFA), Bi-Factor item analyses (bi-factor), and Item Response Modeling analyses (IRM) for the Physical Activity (PA) parenting practices item bank

**Neglect/control PA parenting practices**					
**CFA & Bi-Factor N-618**	**χ**^**2**^	**RMSEA [90% CI]**	**CFI**	**WRMR**	
CFA: Hypothesized 2-factor model	χ^2^(df = 188) = 990, *p* < .05	.083 [.078–.088]	.97	1.34	Adequate fit
Bi-Factor item analysis	χ^2^(df = 168) = 615, *p* < .05	.066 [.060–.071]	.98	0.95	Adequate fit
**IRM** ***N*** **= 618**	**M**^**2**^	**RMSEA [90% CI]**	**CFI**	**SRMR**	**LD / DRF**
IRM: Coercive control	M^2^(df = 111) = 396, p < .05	.065 [.058–.071]	.97	0.42	No LD / No DRF
**Structure PA parenting practices**					
**CFA & Bi-Factor N = 626**	**χ**^**2**^	**RMSEA [90% CI]**	**CFI**	**WRMR**	
CFA: Hypothesized 6-factor model	χ^2^(df = 545) = 4010, *p* < .05	.101 [.098–.104]	.79	2.87	Poor fit
CFA: Modified 5-factor structure **(solution below)**	χ^2^(df = 393) = 1458, *p* < .05	.066 [.062–.069]	.93	1.69	Adequate fit
Bi-Factor item analysis	Not applicable since the correlations among constructs are too low				
**IRM N = 626**	**M**^**2**^	**RMSEA [90% CI]**	**CFI**	**SRMR**	**LD / DRF**
IRM: Nondirective support	M^2^(df = 5) = 28, p < .05	.087 [.058–.119]	.93	0.12	No LD / No DRF
IRM: Supportive expectations	Fit indices not computed when items are less than 10				No LD / No DRF
IRM: Facilitation	Fit indices not computed when items are less than 10				1 LD / No DRF
IRM: Restrict inside PA	Fit indices not computed when items are less than 10				1 LD / No DRF
IRM: Allow unsupervised PA outside	Fit indices not computed when items are less than 10				1 LD / No DRF
**Autonomy promotion PA parenting practices**					
**Model fit**					
**CFA & Bi-Factor N = 626**	**χ**^**2**^	**RMSEA [90% CI]**	**CFI**	**WRMR**	
CFA: Hypothesized 4-factor model	χ^2^(df = 696) = 6269, *p* < .05	.113 [.111–.116]	.79	3.26	Poor fit
CFA: Modified 4-factor structure	χ^2^(df = 316) = 1590, *p* < .05	.080 [.076–.084]	.94	1.89	Adequate fit
Bi-Factor autonomy support	χ^2^(df = 75) = 607, *p* < .05	.106 [.099–.114]	.96	1.33	Adequate fit
**IRM N = 626**	**M**^**2**^	**RMSEA [90% CI]**	**CFI**	**SRMR**	**LD / DRF**
IRM Autonomy support	Fit indices not computed when items are less than 10				2 LD / No DRF
IRM Guided choice	Fit indices not computed when items are less than 10				2 LD / No DRF
IRM Reward	Fit indices not computed when items are less than 10				No LD / No DRF

χ^2^ / M^2^ = Chi-square, *RMSEA* Root Mean Square Error of Approximation, *90% CI* 90% Confidence Interval, where upper 90%CI less than. 10 is indicative of a good fit; *CFI* Comparative Fit Index, where values>.90 to 95 indicative of a good fit; *WRMR* Weighted Room Mean Residual, where values less than 2.0 indicative of a good fit. *SRMR* Standardized Root Mean Residual, where values less than .08 indicative of a good fit, *LD* Local Dependence, *DRF* Differential Response Functioning

**Table 3:** Results from the Confirmatory Factor Analyses (CFA), bi-factor item analyses, and Item Response Modeling (IRM) analyses for the Physical Activity (PA) parenting practices item bank

	CFA		Bi-Factor Item Analysis				Drop code
	Constructs (Cronbach α)	λ	Constructs		λ	I-ECV	
**Neglect/control PA parenting practices domain**							
1 Allow child to stay inside	Permissive (.70)	.83	Coercive control		.70	.47	I-ECV
2 Lack energy to make sure child is active everyday		.56			.48	.88	Poor
3 Allow child not to enroll in sports or activities		.78			.66	.87	
4 Nag or constantly remind child to be active	Pressure (.96)	.70			.69	.72	
5 Threaten / take away privileges for not being active		.62			.62	.84	
6 Guilt child to be PA by telling them he/she is lazy		.90			.91	1.00	
7 Insist child play outside to get child active		.79			.79	.90	
8 Get upset or angry if my child is not active		.88			.89	1.00	
9 Push child hard to improve at sports and activities		.81			.81	.99	
10 Force my child to play outside		.70			.70	.96	
11 Discipline child for refusing to exercise or be active		.91			.91	.99	
12 Promise a sweet or salty treat for being active		.83			.83	.96	
13 Complain when child is not active enough		.83			.83	.98	
14 Say friends will make fun if not better at activities		.91			.91	.97	
15 Insist child participate in organize sports activities		.78			.78	.98	
16 Show people who are overweight to encourage PA		.83			.82	.89	
17 Tell child s/he will gain weight		.76			.75	.85	
18 Tell child will develop diabetes or other disease		.82			.81	.90	
19 Take something or add chore for not being active		.90			.90	.99	
20 Tell child to stop being lazy		.88			.88	1.00	
21 Force child to be active in free time		.88			.88	.98	
**Correlations between constructs**						PE	PR
Permissive (PE)						1.0	
Pressure (PR)						.85	1.0
**Structure PA parenting practices domain**							
22 Participate in any PA with your child	Nondirective support (.86)	.73	NI				
23 Go for walks with your child		.58	NI				
24 Active transportation (walk / bike places) with child		.53	NI				
25 Ask your child to do PA with you		.78	NI				
26 Our family is physically active together		.85	NI				
27 PA is central to what our families does together		.84	NI				
28 Tell my child how much I like PA		–	NI				CFA
29 Talk about my PA with my child		.61	NI				
^a^30 How many times parent did 30 min of PA		.42	NI				
31 Keep track of your child’s PA		.64	NI				
32 Aware of how much PA child engages in		–	NI				CFA
33 Arrange for child to be with friends to be active		.55	NI				
34 Child can easily access sport of PA equipment	Supportive expectation (.76)	–	NI				CFA
35 Make sure child has outside PA/sport equipment		.73	NI				
36 Buy equipment / toys to play outside		.66	NI				
37 Expect that child play outside		.72	NI				
38 Expect that child be active most days of the week		.61	NI				
39 Expect that child be PA through play in free time		.68	NI				
40 Expect family be active together every week		.78	NI				
41 Minutes of expected PA per day		.50	NI				
42 Expect child to enroll in PA outside of school		.59	NI				
43 When school is out, expect child get 60 min of PA		–	NI				CFA
^a^44 f Enroll child in sport or PA during school year	Facilitation (.76)	.77	NI				
^a^45 Take child to sport or PA during school year		.93	NI				LD
^a^46 Enroll child in sport or PA during summer		.58	NI				
^a^47 Take child to sport or PA during summer, d/wk		.67	NI				
^a^48 Miss sport or PA class because no ride		–	NI				CFA
49 Restrict active play inside the home	Restrict inside PA (.70)	.48	NI				
50 Tell child to stop active play for fear of getting hurt		.93	NI				
51 Prevent active play for fear of getting hurt		.78	NI				
52 Enroll child in PA that have risk of minor injury	Allow unsupervised outdoor PA (.73)	–	NI				CFA
^a^53 Limit enrolment in sport/PA during the school year		–	NI				CFA
^a^54 Allow child to play outside alone		.90	NI				
^a^55 Allow child to walk places alone		.97	NI				
^a^56 Allow child to bike alone		.90	NI				LD
^a^57 Allow child to take public transit alone		.59	NI				
**Correlations among constructs**			NS	SE	FA	RI	AU
Nondirective support (NS)			1.0				
Supportive expectations (SE)			.61	1.0			
Facilitation (FA)			.38	.38	1.0		
Restrict inside PA (RI)			.25	.11	.16	1.0	
Allow unsupervised outside PA (AU)			.11	.14	.21	−.02	1.0
**Autonomy promotion PA parenting practices domain**							
58 Encourage PA outside on the weekends	Encourage (.90)	.58	Autonomy support		.55	.96	
59 Encourage to go places to be active		–			–		CFA
60 Encourage walking & biking in the neighbourhood		–			–		CFA
61 Suggest walking or biking to get to places		–			–		CFA
62 Say positive things to motivate child to be active		–			–		CFA
63 Help find ways for your child to be PA in free time		.80			.69	.74	LD
64 Tell child that PA is fun		–			–		CFA
65 Tell my child he/she will make friends by being PA		–			–		CFA
66 Discuss benefits of PA with your child		.85			.77	.85	
67 Tell your child you like it when they are active		.82			.73	.82	
68 Use role models to encourage PA		–			.51	.81	
69 Remind your child to be PA in their free time		.81			.66	.53	I-ECV
70 Set challenges to encourage more activity		–			–		CFA
71 Remind my child to be play more actively		.42			.26	.25	I-ECV
72 Teach child new or different games to be active		–			–		CFA
73 Volunteer or organize sports or PA activities	Parent involvement (.89)	–			–		CFA
74 Encourage your child to talk about PA		–			–		CFA
75 Talk about child’s sports or PA participation		.74			.61	.66	
76 Tell child is doing well in PA		.88			.74	.68	LD
77 Watch child’s sports practices		.67			.45	.34	I-ECV
78 Teach child a sport or PA		.60			.61	.99	
79 Tell child you are proud of their PA participation		.91			.80	.78	
80 Praise child for participating in PA or sport		.90			.82	.83	
81 Watch child’s sports games or performances		.47			.26	.21	I-ECV
82 Coach child’s sports or activities		–					CFA
83 Find it stimulating to hear about child’s PA		.60			.48	.64	
^a^84 Child provides input on the types of PA like to do	Guided choice (.88)	.81	NI		–		
^a^85 Involve child in picking what activities to enroll in		.77	NI		–		
^a^86 Child given PA choices from which s/he can pick		.80	NI		–		
^a^87 We pick what activities my child will do together		.77	NI		–		
^a^88 Ask child to decide when active in free time		.64	NI		–		
^a^89 Agree on when child should be active in free time		.69	NI		–		
^a^90 Encourage child to come up with a PA plan		.64	NI		–		LD
^a^91 Calmly discuss when child should be active		.65	NI		–		LD
^a^92 Child can choose activities we do as a family		.71	NI		–		
93 Reward child for being physically active	Reward (.92)	.90	NI		–		
94 Reward child for PA accomplishments		–	NI		–		CFA
95 Reward child for trying hard		.88	NI		–		
96 Reward child for participating in PA classes		.95	NI		–		
**Correlations among constructs**			EN		PI	GC	RE
Encourage (EN)			1.0				
Parent involvement (PI)			.75		1.0		
Guided choice (GC)			.35		.46	1.0	
Reward (RE)			.40		.29	.16	1.0

^a^ Items that were administered at Wave 2 (N ranged from 459 to 475, except for item 48 where *N* = 386)

Drop code: *CFA* Dropped from the Confirmatory Factor Analyses as it was not loading on the factor or identified as correlating with other factors from the modification indices, *DRF* Deleted since item is not invariant (significant Differential Response Functioning), I-ECV (explained common variance for a single item) is less than .50 I-ECV, *LD* LD Local dependence; and Poor = Conceptually dropped as thought to be a poor indicator of the construct

#### Invariance properties

DIF and DRF were assessed on the remaining 19 items and none of the items exhibited any significant DIF or DRF by parents’ sex, income, or ethnicity.

#### Efficiency

In total, the coercive control construct includes 19 items and the overall IRM empirical reliability of the bank is .95. From the CAT simulations, it was estimated that 7 items are needed to maintain the reliability of the total scores at .80 with the correlation between the short and long form equal to .95. Table 4 shows the items retained in the short form.

**Table 4:** Physical activity (PA) parenting practices item bank – full list of items by domain and list of items included in the short form

Constructs # items IRM reliability^a^	Items		Short form
**Neglect/control PA parenting practices domain**			
Coercive control 19 items (.94)	3	My child can convince me to not enroll him/her in any physical activity or sport classes during the year	
	4	I have to nag or constantly remind my child to be physically active in his/her free time.	√
	5	I threaten to take away privileges (e.g., TV or video game times) if my child does not spend time being physically active in his/her free time	
	6	I try to guilt my child to be more physically active by telling him/her that s/he has been lazy	
	7	The only way I can get my child to play outside is by insisting that my child goes outside	√
	8	My child knows that I get upset and angry at him/her if s/he is not participating in physical activity in his/her free time	
	9	To help my child improve at sports or physical activity, I have to push my child hard	√
	10	When the weather allows, I force my child to play outside even if s/he does not feel like it	√
	11	I discipline my child for refusing to exercise or being inactive in his/her free time	
	12	To encourage my child to be physically active, I promise a sweet or salty treat (e.g., dessert) if s/he is active	
	13	I complain to my child when s/he is not active enough in order to get him/her to be more active	
	14	To get my child to practice his/her activities, I often say “your friends will make fun of you if you do not get better at your activities (e.g., sport, dance)	√
	15	I insist that my child participates in organized sports or physical activities instead of playing with his/her friends	
	16	I show my child people who are unhealthy (overweight) to get him or her to be more physically active	
	17	I tell my child s/he will gain weight if s/he doesn’t exercise	
	18	I tell my child s/he will get diabetes or other diseases if s/he is not physically active on a regular basis	
	19	I take something away (no dessert or TV) or add an additional chore (clean up toys) if my child refuses to take part in physical activity or sports	
	20	To make my child do more physical activity in his/her free time, I tell him/her to stop being lazy	√
	21	The only way I get my child to exercise or be physically active in his/her free time is by forcing him/her to be active	√
**Structure PA parenting practices domain**			
Nondirective support 10 items (.89)	22	Participate in any physical activity (such as playing ball or sports) with your child	√
	23	Go for walks with your child	
	24	Walk or bike with your child to go to places that are near your home (a few minutes away) even though it would be quicker to drive	
	25	Ask your child to exercise or be physically active with you	√
	26	Our family is physically active together	√
	27	Participation in physical activity and sports is central to what our family does together	
	29	I talk about my physical activity with my child	
	30	Do at least 30 min of physical activity or exercise (e.g., walking, cycling, or playing a sport) on your own or with others? ^d^	
	31	Keep track (in your head or writing down) whether your child did 60 min physical activity or exercise every day ^d^	
	33	Arrange for your child to be with friends that would encourage your child to be physically active	
Supportive expectation 8 items (.85)	35	I make sure my child has the physical activity or sport equipment to use when s/he wants to play outside (like soccer balls, basketballs, or active outdoor toys)	√
	36	I often buy active or outdoor physical activity equipment or toys to encourage my child to play outside	
	37	When the weather allows, I have expectations that my child should play outside	√
	38	I believe that children should participate in some form of physical activity or sports on most days of the week	
	39	I have expectations that my child should get physical activity through play in his/her free time	√
	40	I have expectations that my family should be physically active together every week	
	41	I have expectations that my child must be physically active every day for about	
	42	During the school year, I expect my child to enroll in physical activities or sports outside of the school day, at least …	
Facilitation 3 items (.79)	44	During the SCHOOL YEAR, I enroll my child in organized sport or physical activity classes (e.g., swimming lessons, dance, karate, soccer, or other)	√
	46	When school is out in the SUMMER, I find ways for my child to be physically active by enrolling him/her in summer activities (including sport related summer camps)	√
	47	When school is out in the SUMMER, how many days per week did you typically spend taking your child to his/her sport or physical activity classes or practices (excluding summer camps)	√
Restrict inside PA 3 items (.80)	49	How often do you restrict active play (e.g., ball games, running, wrestling) inside your home	√
	50	How often do you tell your child to stop playing too actively because someone may get hurt if s/he continues the activity	√
	51	How often do you prevent your child from playing actively for fear of someone getting hurt	√
Allow unsurpervised outdoor PA 3 items (.89)	54	Do you let your child play outside on his/her own without direct adult supervision?	√
	55	Do you let your child walk to places on his/her own?	√
	57	Do you let your child take public transportation to places on his/her own?	√
**Autonomy promotion PA parenting practices domain**			
Autonomy support 9 items (.91)	58	On the weekends, I encourage my child to play outside when the weather allows	
	66	Discuss the benefits of being active with your child without making your child feel bad	
	67	Tell your child that you like it when s/he spends time outdoors being active	
	68	I show my child examples of role models (people who are active) that my child can relate with to encourage him/her to be physically active	
	75	Make your child’s sport or physical activity participation a topic of family conversation	
	78	Spend time teaching your child how to play a sport or learn a physical activity skill	
	79	Tell your child that you are proud of him/her for participating in any physical activity or something to do with sports	√
	80	Praise your child for being physically active or for participating in sports or physical activity classes	√
	83	I find it stimulating to hear my child talk about the progress s/he is making in learning a new sport or physical activity skill	
Guided Choice 7 items (.88)	84	I asked my child to let me know what activities s/he would like to do	√
	85	I involved my child in deciding which physical activity or sports s/he is enrolled in	√
	86	I provided my child with choices about the physical activity s/he does	√
	87	I allowed my child to pick the types of physical activity/sports we do together	
	88	I asked my child to decide when s/he could be active in his/her free time.	
	89	When I discuss with my child when s/he should be active, we can quickly agree on a solution we are both happy with	
	92	I allowed my child to choose the physical activity/sports we do as a family (whether we go for a walk, hike, bike ride, or play an active game).	√
Reward 3 items (.88)	93	I give my child a small reward (e.g., sticker, badge, or take my child to a movie) when s/he Is being physically active in his/her free time	√
	95	I give my child a small reward (e.g., sticker, badge, or take my child to a movie) when s/he tries hard at his/her physical activity or sport	√
	96	I give my child a small reward (e.g., sticker, badge, or take my child to a movie) when s/he participates in organized sports or physical activity classes	√

IRM reliability = Empirical reliability computed from Item Response Modeling (IRM) which takes into account the ordinal nature of the data

^a^The IRM reliability for the short form is fixed at .80 for these constructs

√ = Item included in the short form

### Structure domain of PAPP

#### Structural validity

As shown in Table 2, the hypothesized 6-factor CFA for the structure domain did not fit the data. In the revised solution seven items were deleted to eliminate items that had high correlated errors or did not load as hypothesised and included the following changes: a) the co-participation, modeling, and monitoring constructs were combined into a new construct labelled nondirective support; b) the expectations and some items from the facilitation construct were regrouped into supportive expectations; c) some of the remaining items from the facilitation construct remained on that construct; and d) the restriction construct was split into two constructs, namely restricting inside PA and allowing unsupervised outside PA. As shown in Table 2, this revised 5-fator solution had an adequate fit. As none of the constructs were highly correlated (all *r*’s were less than .70) (see Table 3), the analyses proceeded directly to the IRM. From the IRM analyses, three items were deleted as they had high local dependence which suggested that their content was redundant with other items.

#### Invariance properties

All items for the structure constructs were invariant by parents’ sex, income, and ethnicity.

***Efficiency*** was assessed for two constructs, namely nondirective support and supportive expectations, as the IRM empirical reliability was greater than .80 and these constructs included more than five items. From the CAT simulations, it was estimated that the reliability of the total score can remain at .80 if three items are retained for the nondirective support construct and three items are retained for the supportive expectations construct (See Table 4 for list of items). The correlations between the long and short forms were .90 and .76 for the nondirective and support constructs, respectively.

### Autonomy support domain of PAPP

#### Structural validity

As shown in Table 2, the hypothesized 4-factor solution for the autonomy promotion domain of PAPP did not fit the data. However, a 4-factor solution that eliminated 12 items and moved the praise items to the involvement and encouragement constructs with the reward items forming a reward construct had an adequate overall fit. Correlation among constructs ranged from .16 to .75. As the involvement and encouragement constructs were highly correlated (.75), a common bi-factor item analysis was run. The overall fit of the confirmatory bi-factor solution was acceptable and supported combining the encourage and involvement constructs to measure a more general construct renamed autonomy support (see the solution in Table 3). The IRM analyses further assessed the unidimensionality of the resulting three constructs that assessed autonomy promotion. This process led to the deletion of more items that were redundant as they showed some local dependence with other items on the constructs.

#### Invariance properties

The DRF analyses found all items were invariant by parents’ sex, income, and ethnicity.

***Efficiency*** was assessed for two constructs, namely autonomy promotion and guided choice as both had IRM empirical reliability greater than .80 and included 5 or more items. From the CAT simulation, the reliability of the scores can be maintained at .80 with two items for the autonomy promotion construct and with 4 items for the guided choice constructs (as indicated in Table 4). The correlation between the short and long forms was .86 and .95 for the autonomy support and guided choice, respectively.

### Overview of results and alignment with the conceptual framework

**Fig. 2:**
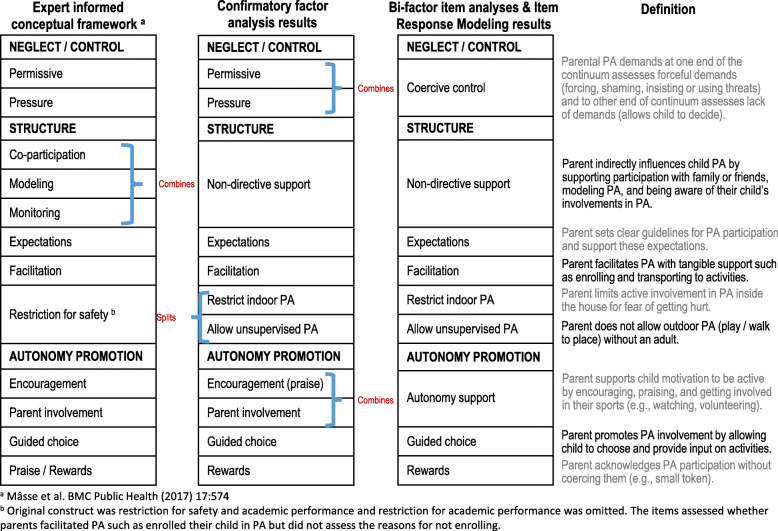
Expert informed Physical Activity (PA) conceptual framework and its alignment with the results

Figure 2 shows the extent to which the findings align with the expert informed conceptual framework.16) The CFA results directly aligned for the neglect/control domain of parenting, are quite similar for the autonomy promotion domain of parenting and resulted in some changes for the structure domain of parenting. As the analyses progressed to identify whether some constructs could be collapsed to measure more general constructs, the alignment with the guiding conceptual framework began to diverge (see how the bi-factor and IRM results modified the structure). Fig. 2 also presents the operational definitions of the PAPP item bank constructs.

## Discussion

This is the first study that utilizes a conceptual framework developed by an international panel of experts to achieve parsimony and gain efficiency in measuring PA parenting practices of 5–12 year-old children. The advanced psychometric analyses were anchored to the expert panel conceptual model to inform the structural validity of scores derived from the PAPP item bank. The underlying conceptual framework of the PAPP item bank was mainly supported by the CFAs. Some changes were made to the underlying structure based on the CFAs including: a) combining smaller constructs into one general construct (for example, combining the modeling, co-participation, and monitoring constructs of the structure domain into a general construct assessing nondirective support); or b) splitting a construct into two smaller constructs (for example, splitting the restrict for safety reason construct into a construct that assessed indoor PA restriction and another one that assessed allowance for outside PA). While the CFAs supported the structural validity of 11 constructs, the bi-factor item analyses and IRM analyses supported collapsing correlated constructs into more general constructs. These analyses further reduced the number of constructs measured by the PAPP item bank into nine constructs with internal consistency ranging from .79 to .94 and a total of 65 items. As seven of the PAPP constructs has reliability greater than .80, CAT simulations could further reduce the number of items for those constructs while maintaining high reliability. The CAT simulations found that the final nine constructs can be efficiently assessed with as a little as 31 items (see Table 4 and a full list of items is provided in Appendix A). Overall, the PAPP item bank has excellent psychometric properties and provides an efficient way to assess PA parenting practices.

The PAPP item bank addresses an identified need to align PA parenting practice measures with the parenting literature as a way to bring more rigour and consistency in operationalizing the PA parenting practices constructs [9, 17]. To achieve this, the conceptual framework which served as the foundation for the construct validity analyses: a) integrated knowledge of the parenting literature to provide assessment of the main domains of parenting (i.e., neglect/control, structure, and autonomy promotion) [16], b) consolidated knowledge from existing research, and c) incorporated inductive inquiry of parents to ensure the PAPP constructs included strategies used by parents [21]. The expert-informed conceptual framework provided a structure which we expected to validate with the CFAs. For the neglect/control and autonomy promotion domains of parenting, there was strong alignment between the CFAs and the expert-informed conceptual framework. In the original conceptual framework, praise and rewards were combined as one construct but the CFAs supported combining the praise items with encouragement which makes sense as praise is a form of encouragement. For the structure domain of parenting, there were more differences between the CFAs and the expert-informed conceptual framework than anticipated. The co-participation, modeling, and monitoring constructs were so highly correlated that they ended being combined through the CFA analyses and formed a more general construct. The CFAs did not support analyzing these three constructs separately. It makes sense that modeling and co-participation would be highly correlated as co-participation is likely higher among those who are highly active parents. Also, it is likely that active parents who value PA tend to be more aware of their child’s level of PA and monitor whether they should engage in PA with their child. Combining these separate constructs into a general construct that measures non-directive support is a departure from what has been previously done but needed to account for the overlap in these concepts. Finally, the CFAs found that the content for the restriction for safety split into two constructs. Conceptually the split regrouped items that focused on unsupervised outdoor activities and the other focused on injuries associated with playing roughly indoor. Overall, the skeleton of the expert-informed conceptual framework can be mapped out onto the CFAs as when a new construct emerged it still combined elements of the original conceptual framework without loosing any content or it added nuances by splitting a construct into two.

Interestingly, the two constructs within the neglect/control domain of parenting, namely the permissive and pressure constructs combined into one global construct. At the time the conceptual framework was developed there were extensive discussions as to whether permissive should be listed under the control domain of parenting and when we decided to so we renamed this domain of parenting as neglect/control. In the conceptual framework, pressure was defined as including the “coercive” component of control based on Baumrind’s definition and Grolnick and Pomerantz’s operationalization of control and permissive was defined as “lack of willingness to act as a socializing agent” which aligns with Darling and Steinberg’s operationalization of control [11, 19, 34]. While permissive and pressure have been considered as distinct constucts in the PA literature, there is some support from the parenting literature to align permissive and pressure onto a control continuum [11]. In fact, the bi-factor and IRM results suggest that as operationalized in the PAPP item bank the permissive and pressure constructs are on the same continuum and measure one dimension. This represents an important modification but one that aligns with the existing parenting literature.

Measurement of general constructs is not something that has been typically done in the PA parenting field but has occurred in fields that have integrated advanced psychometric methods in instrument development, such as outcomes research [18, 35]. This process is more easily understood when collapsing of constructs arises from the CFAs, meaning the structure needs to be simplified in order for the solution to fit the data. As discussed above, this was the case for the nondirective support construct as the CFA regrouped three constructs that have generally been independently assessed (co-participation, modeling, and monitoring). The bi-factor and IRM analyses further supported collapsing some of the constructs found in the CFAs into more general constructs. While some researchers may opt to use the results of the CFA analyses, it is worth highlighting the collapsing into more general constructs occurred because these constructs were found to be highly correlated in the CFA analyses. Furthermore, the bi-factor and IRM analyses further tested whether these constructs are unidimensional, meaning it assessed whether these items measure a single continuum. The combining of constructs into more general constructs represent a departure from the expert-informed conceptual framework. However, it is important to note that the process used to develop the conceptual framework did not discuss how the PAPP constructs may be combined to measure more general constructs. Therefore, the conceptual framework was primarily used to guide the CFAs and the analyses beyond the CFAs served to expand our conceptual understanding and move our thinking into assessing more general PAPP constructs. This represents a departure for the PA literature but an important one to move the field of PA parenting forward. As the process to identify general constructs was analytically informed, further studies should cross-validate the stability of these newly identified constructs.

It is also worth noting that the analysis prioritized collapsing general constructs within the main domain of parenting to ensure the analyses aligned with the parenting literature and the conceptual framework that guided this work. The correlations among the final constructs ranged in absolute value from .01 to .66 and as such did not meet the rule to proceed with a bi-factor analysis correlation because the correlations were not sufficiently high (>.70). This further highlights that the analytical process reduced the conceptual framework into the smallest number of constructs.

To optimize the use of the calibrated item bank, the next steps are to take advantage of the features associated with having a calibrated item bank [35]. This includes: a) allowing researchers to either select or supplement the constructs with items that are relevant for their study while ensuring that their results can be compared with other studies; b) administering the item bank with CAT as this process would optimize the efficiency of the administration and precision of the total scores; and c) using simulated CAT to develop short forms [35]. The first two features associated with item banking will need to be addressed in continued extension of this program of research. Whereas, the last feature was developed in this paper with efficient short forms developed. The CAT simulations identified that the nine PAPP constructs can be efficiently assessed with 31 items from the existing item pool of 65 items, highlighting the utility of using advanced psychometric methods to develop measures of PAPP that will decrease participant burden. In addition, future extension of this program of research needs to further assess the construct validity of the PAPP item bank to determine whether the PAPP constructs predict child’s PA behaviours. This can help focus which constructs are important to measure in future studies.

It is important to highlight that while short forms have some advantages such as decreased participant burden the short form should be cautiously used. The short form does not capture the full breadth of the constructs and as such should be interpreted cautiously. As the short forms are highly correlated with the long forms (correlations ranging from .76 to .95), the short forms are expected to yield very similar rankings to the long forms and as such can provide good indicators of parenting practices for the overall constructs. The short forms are likely better suited for correlational studies than for interventional studies where capturing the full breadth of the content is important. In addition, in the intervention context it is important to use scales that are sensitive to change and this may be less likely with the short forms.

The psychometric validation must be interpreted in light of the limitations of this study. First, the sample used for the item calibration was recruited from an English speaking web-based panel company in Canada and as such non-English speaking parents were excluded as this was a study requirement. Second, as the calibration was conducted in a Canadian sample, the extent to which the properties found in this study are stable across other populations remains unknown. Third, while invariance of item parameters was examined across ethnicity, these analyses were limited to comparing Caucasians versus all other ethnicities. Fourth, our sample size precluded us from splitting our sample into two samples so that the findings could be cross-validated. Therefore, future studies will need to further assess the psychometric properties of the item bank in larger, more varied samples to obtain measures that are stable across various ethnic groups and especially focus on cross-validating the structure of the general constructs.

In conclusion, the psychometric validation supported a simplified version of the PAPP conceptual framework for 5–12 year-old children, which was initially endorsed by 24 experts from 6 countries. The PAPP item bank resulted in nine constructs that assessed three main domains of PA parenting practices namely neglect/control, structure, and autonomy promotion. The CAT simulations demonstrated the potential of using calibrated item banking to develop short forms that have adequate reliability and resulted in the development of shorter measures to assess these nine construct (31 items instead of 65 items).

## Supplementary information

Additional file 1. (DOC 61.6 kb)

## Data Availability

Please contact LCM at lmasse@bcchr.ubc.ca for any data requests or study materials.
