# Supplementary material for: The physical activity parenting practices (PAPP) item Bank: a psychometrically validated tool for improving the measurement of physical activity parenting practices of parents of 5–12-year-old children
Source: Int J Behav Nutr Phys Act. 2020 Nov 4;17:134. doi: 10.1186/s12966-020-01036-0 (PMC7641851; doi:10.1186/s12966-020-01036-0)
Supplement: Supplementary file 1 — (DOC 61.6 kb) [file 12966_2020_1036_MOESM1_ESM.docx]

**Appendix A**

**Physical Activity Parenting Practices**

**Item Bank Long Form (65 items)**

***The next questions ask whether you have done the following activities in the past MONTH. The “Never, this has not been possible lately” may be the most appropriate answer if you did not do this lately or your child was not physically active***

**In the PAST MONTH, how often did you… (Answer for yourself only). *Choose one for each row.***

Q1. Participate in any physical activity (such as playing ball or sports) with your child ***nondirective support_22***

Q2. Go for walks with your child ***nondirective support_23***

Q3. Walk or bike with your child to go to places that are near your home (a few minutes away) even though it would be quicker to drive ***nondirective support_24***

Q4. Ask your child to exercise or be physically active with you ***nondirective support_25***

Q5. Keep track (in your head or writing down) whether your child did 60 minutes of physical activity or exercise every day ***nondirective support_31***

Q6. Arrange for your child to be with friends that would encourage your child to be physically active ***nondirective support_33***

Q7. Discuss the benefits of being active with your child without making your child feel bad ***autonomy support_66***

Q8. Tell your child that you like it when s/he spends time outdoors being active ***autonomy support_67***

Q9. Make your child’s sport or physical activity participation a topic of family conversation ***autonomy support_75***

Q10. Spend time teaching your child how to play a sport or learn a physical activity skill ***autonomy support_78***

Q11. Tell your child that you are proud of him/her for participating in any physical activity or something to do with sports ***autonomy support_79***

Q12. Praise your child for being physically active or for participating in sports or physical activity classes ***autonomy support_80***

Never – this has not been possible lately

1-2 times per month

3-4 times per month

2-3 times per week

4 or more times per week

***The next questions ask the extent to which you do these activities with your child or as a family. Select the best answer for you. Choose one for each row.***

Q13. My child can convince me to not enroll him/her in any physical activity or sport classes during the year. ***coercive control_3***

Q14. I have to nag or constantly remind my child to be physically active in his/her free time. ***coercive control_4***

Q15. I threaten to take away privileges (e.g., TV or video game times) if my child does not spend time being physically active in his/her free time. (***coercive control_5***

Q16. I try to guilt my child to be more physically active by telling him/her that s/he has been lazy.  ***coercive control_6***

Q17. The only way I can get my child to play outside is by insisting that my child goes outside. ***coercive control_7***

Q18. My child knows that I get upset and angry at him/her if s/he is not participating in physical activity in his/her free time. ***coercive control_8***

Q19. To help my child improve at sports or physical activity, I have to push my child hard. ***coercive control_9***

Q20. When the weather allows, I force my child to play outside even if s/he does not feel like it. ***coercive control_10***

Q21. I discipline my child for refusing to exercise or being inactive in his/her free time. ***coercive control_11***

Q22. To encourage my child to be physically active, I promise a sweet or salty treat (e.g., dessert) if s/he is active. ***coercive control_12***

Q23. I complain to my child when s/he is not active enough in order to get him/her to be more active. ***coercive control_13***

Q24. To get my child to practice his/her activities, I often say “your friends will make fun of you if you do not get better at your activities (e.g., sport, dance). ***coercive control_14***

Q25. I insist that my child participates in organized sports or physical activities instead of playing with his/her friends. ***coercive control_15***

Q26. I show my child people who are unhealthy (overweight) to get him or her to be more physically active. ***coercive control_16***

Q27. I tell my child s/he will gain weight if s/he doesn’t exercise. ***coercive control_17***

Q28. I tell my child s/he will get diabetes or other diseases if s/he is not physically active on a regular basis. ***coercive control_18***

Q29, I take something away (no dessert or TV) or add an additional chore (clean up toys) if my child refuses to take part in physical activity or sports. ***coercive control_19***

Q30. To make my child do more physical activity in his/her free time, I tell him/her to stop being lazy. ***coercive control_20***

Q31. The only way I get my child to exercise or be physically active in his/her free time is by forcing him/her to be active. ***coercive control_21***

Q32. Our family is physically active together. ***nondirective support_26***

Q33. Participation in physical activity and sports is central to what our family does together. ***nondirective support_27***

Q34. I talk about my physical activity with my child. ***nondirective support_29***

Q35. On the weekends, I encourage my child to play outside when the weather allows. ***autonomy support_58***

Q36. I show my child examples of role models (people who are active) that my child can relate with to encourage him/her to be physically active. ***autonomy support_68***

Q37. I find it stimulating to hear my child talk about the progress s/he is making in learning a new sport or physical activity skill. ***autonomy support_83***

Never

Rarely

Sometimes

Often

Very often

***Choose one answer for each row.***

Q38. How often do you restrict active play (e.g., ball games, running, wrestling) inside your home? ***restrict inside_49***

Q39. How often do you tell your child to stop playing too actively because someone may get hurt if s/he continues the activity? ***restrict inside_50***

Q40. How often do you prevent your child from playing actively for fear of someone getting hurt? ***restrict inside_51***

Q41. How often do you give your child a small reward (e.g., sticker, badge, or take my child to a movie) when s/he is being physically active in his/her free time. ***reward_93***

Q42. How often do you give your child a small reward (e.g., sticker, badge, or take my child to a movie) when s/he Tries hard at his/her physical activity or sport ***reward_95***

Q43. How often do you give your child a small reward (e.g., sticker, badge, or take my child to a movie) when s/he participates in organized sports or physical activity classes. ***reward_96***

Never, I reward for other things

Rarely

About half the time

Often

Most of the time

**How much do you agree or disagree with each of the following? Select the most appropriate answer.**

Q44. I make sure my child has the physical activity or sport equipment to use when s/he wants to play outside (like soccer balls, basketballs, or active outdoor toys). ***supportive expectation_35***

Q45. I often buy active or outdoor physical activity equipment or toys to encourage my child to play outside. **supportive expectation_36**

Q46. When the weather allows, I have expectations that my child should play outside. ***supportive expectation_37***

Q47. I believe that children should participate in some form of physical activity or sports on most days of the week. ***supportive expectation_38***

Q48. I have expectations that my child should get physical activity through play in his/her free time. ***supportive expectation_39***

Q49. I have expectations that my family should be physically active together every week. ***supportive expectation_40***

Strongly disagree

Disagree

Neutral

Agree

Strongly agree

Q50. I have expectations that my child must be physically active every day for about… *Choose one.* ***supportive expectation_41***

20 minutes per day

30 minutes per day

45 minutes per day

60 minutes per day or more

I don’t have an expectation that my child be physically active

Q51. During the school year, I expect my child to enroll in physical activities or sports outside of the school day, at least... ***supportive expectation_42***

No expectations – my child decides this

Less than once a week

1 day a week

2 days a week

3 days a week

4 days a week

5 days a week

6 days a week

7 days a week

**The next questions ask about your child’s involvement in physical activity over the past YEAR.**

Q52. During the SCHOOL YEAR, I enroll my child in organized sport or physical activity classes (e.g., swimming lessons, dance, karate, soccer, or other). *Choose one* ***facilitation_44***

No, my child was not enrolled

Yes, my child was enrolled for half of the school year

Yes, my child was enrolled for most of the school year

Q53. When school is out in the SUMMER, I find ways for my child to be physically active by enrolling him/her in summer activities (including sport related summer camps). *Choose one.* ***facilitation_46***

No, my child is not typically enrolled in summer activities

Yes, for part of the summer

Yes, for most of the summer

Q54. When school is out in the SUMMER, how many days per week did you typically spend taking your child to his/her sport or physical activity classes or practices (excluding summer camps)? *Choose one* ***facilitation_47***

None – my child was not enrolled in any weekly classes or practices

1 day a week

2 days a week

3 days a week

4 or more days a week

Q55. Do you let your child walk to places on his/her own? *Choose one.* ***allow unsupervised outdoor PA_55***

No, my child cannot go to places without an adult

Yes, but within our block

Yes, but within 2 to 3 blocks away from our home

Yes, but within our neighbourhood

Yes, outside of our neighbourhood

Q56. Do you let your child play outside on his/her own without direct adult supervision? *Choose one.* ***allow unsupervised outdoor PA_54***

No, my child must be supervised when outside

Yes, but only around our home/yard

Yes, but within our block

Yes, but within 2 to 3 blocks away from our home

Yes, but within our neighbourhood

Yes, outside of our neighbourhood

Q57. Do you let your child take public transportation to places on his/her own? *Choose one.* ***allow unsupervised outdoor PA_57***

No

Yes

**The next questions ask whether you have done the following activities in the past YEAR.**

**In the past YEAR, … Choose one for each row.**

Q58. I asked my child to let me know what activities s/he would like to do. ***guided choice_84***

Q59. I involved my child in deciding which physical activity or sports s/he is enrolled in. ***guided choice_85***

Q60. I provided my child with choices about the physical activity s/he does. ***guided choice_86***

Q.61, allowed my child to pick the types of physical activity/sports we do together. ***guided choice_87***

Q62. I allowed my child to choose the physical activity/sports we do as a family (whether we go for a walk, hike, bike ride, or play an active game). ***guided choice_92***

Not relevant, child currently NOT active

Never, I prefer doing this myself

Rarely

Sometimes

Often

Most of the time

**The next questions ask whether you have done the following activities in the past YEAR. If you have not done the following because your child is not mature enough, select “Never”. In the past YEAR, … *Choose one for each row.***

Q63. I asked my child to decide when s/he could be active in his/her free time. ***guided choice_88***

Q64. When I discuss with my child when s/he should be active, we can quickly agree on a solution we are both happy with. ***guided choice_89***

Not relevant, child currently NOT active

Never

Rarely

Sometimes

Often

Most of the time

**The next questions ask about your involvement in physical activity.**

Q65. In the past MONTH, how many times did you (the parent) do at least 30 minutes of physical activity or exercise (e.g., walking, cycling, or playing a sport) on your own or with others? *Choose one.* ***nondirective support_30***

Never – This has not been possible lately

1-2 times per month

3-4 times per month

2-3 times per week

4 or more times per week

**SCORING**

Scoring for the long form by averaging the items for each construct as follow:

***CONTROL PHYSICAL ACTIVITY PARENTING PRACTICES DOMAIN***

- Coercive control (19 items)

***STRUCTURE PHYSICAL ACTIVITY PARENTING PRACTICES DOMAIN***

- Non directive support (10 items)
- Supportive expectation (8 items)
- Facilitation (3 items)
- Restrict inside physical activity (3 items)
- Allow unsupervised physical activity (3 items)

***AUTONOMY PHYSICAL ACTIVITY PARENTING PRACTICES DOMAIN***

- Autonomy support (9 items)
- Guided choice (7 items)
- Reward (3 items)

Inquiries about the instrument should be sent Dr. L.C. Mâsse at [lmasse@bcchr.ubc.ca](mailto:lmasse@bcchr.ubc.ca)

**Physical Activity Parenting Practices**

**Item Bank Short Form (31 items)**

***The next questions ask whether you have done the following activities in the past MONTH. The “Never, this has not been possible lately” may be the most appropriate answer if you did not do this lately or your child was not physically active***

**In the PAST MONTH, how often did you… (Answer for yourself only). *Choose one for each row.***

Q1. Participate in any physical activity (such as playing ball or sports) with your child ***nondirective support_22***

Q2. Ask your child to exercise or be physically active with you ***nondirective support_25***

Q3. Tell your child that you are proud of him/her for participating in any physical activity or something to do with sports ***autonomy support_79***

Q4. Praise your child for being physically active or for participating in sports or physical activity classes ***autonomy support_80***

Never – this has not been possible lately

1-2 times per month

3-4 times per month

2-3 times per week

4 or more times per week

***The next questions ask the extent to which you do these activities with your child or as a family. Select the best answer for you. Choose one for each row.***

Q5. I have to nag or constantly remind my child to be physically active in his/her free time. ***coercive control_4***

Q6. The only way I can get my child to play outside is by insisting that my child goes outside. ***coercive control_7***

Q7. To help my child improve at sports or physical activity, I have to push my child hard. ***coercive control_9***

Q8. When the weather allows, I force my child to play outside even if s/he does not feel like it. ***coercive control_10***

Q9. To get my child to practice his/her activities, I often say “your friends will make fun of you if you do not get better at your activities (e.g., sport, dance). ***coercive control_14***

Q10. To make my child do more physical activity in his/her free time, I tell him/her to stop being lazy. ***coercive control_20***

Q11. The only way I get my child to exercise or be physically active in his/her free time is by forcing him/her to be active. ***coercive control_21***

Q12. Our family is physically active together. ***nondirective support_26***

Never

Rarely

Sometimes

Often

Very often

***Choose one answer for each row.***

Q13. How often do you restrict active play (e.g., ball games, running, wrestling) inside your home? ***restrict inside_49***

Q14. How often do you tell your child to stop playing too actively because someone may get hurt if s/he continues the activity? ***restrict inside_50***

Q15. How often do you prevent your child from playing actively for fear of someone getting hurt? ***restrict inside_51***

Q16. How often do you give your child a small reward (e.g., sticker, badge, or take my child to a movie) when s/he is being physically active in his/her free time. ***reward_93***

Q17. How often do you give your child a small reward (e.g., sticker, badge, or take my child to a movie) when s/he Tries hard at his/her physical activity or sport ***reward_95***

Q18. How often do you give your child a small reward (e.g., sticker, badge, or take my child to a movie) when s/he participates in organized sports or physical activity classes. ***reward_96***

Never, I reward for other things

Rarely

About half the time

Often

Most of the time

**How much do you agree or disagree with each of the following? Select the most appropriate answer.**

Q19. I make sure my child has the physical activity or sport equipment to use when s/he wants to play outside (like soccer balls, basketballs, or active outdoor toys). ***supportive expectation_35***

Q20. When the weather allows, I have expectations that my child should play outside. ***supportive expectation_37***

Q21. I have expectations that my child should get physical activity through play in his/her free time. ***supportive expectation_39***

Strongly disagree

Disagree

Neutral

Agree

Strongly agree

**The next questions ask about your child’s involvement in physical activity over the past YEAR.**

Q22. During the SCHOOL YEAR, I enroll my child in organized sport or physical activity classes (e.g., swimming lessons, dance, karate, soccer, or other). *Choose one* ***facilitation_44***

No, my child was not enrolled

Yes, my child was enrolled for half of the school year

Yes, my child was enrolled for most of the school year

Q23. When school is out in the SUMMER, I find ways for my child to be physically active by enrolling him/her in summer activities (including sport related summer camps). *Choose one.* ***facilitation_46***

No, my child is not typically enrolled in summer activities

Yes, for part of the summer

Yes, for most of the summer

Q24. When school is out in the SUMMER, how many days per week did you typically spend taking your child to his/her sport or physical activity classes or practices (excluding summer camps)? *Choose one* ***facilitation_47***

None – my child was not enrolled in any weekly classes or practices

1 day a week

2 days a week

3 days a week

4 or more days a week

Q25. Do you let your child walk to places on his/her own? *Choose one.* ***allow unsupervised outdoor PA_55***

No, my child cannot go to places without an adult

Yes, but within our block

Yes, but within 2 to 3 blocks away from our home

Yes, but within our neighbourhood

Yes, outside of our neighbourhood

Q26. Do you let your child play outside on his/her own without direct adult supervision? *Choose one.* ***allow unsupervised outdoor PA_54***

No, my child must be supervised when outside

Yes, but only around our home/yard

Yes, but within our block

Yes, but within 2 to 3 blocks away from our home

Yes, but within our neighbourhood

Yes, outside of our neighbourhood

Q27. Do you let your child take public transportation to places on his/her own? *Choose one.* ***allow unsupervised outdoor PA_57***

No

Yes

**The next questions ask whether you have done the following activities in the past YEAR.**

**In the past YEAR, … Choose one for each row.**

Q28. I asked my child to let me know what activities s/he would like to do. ***guided choice_84***

Q29. I involved my child in deciding which physical activity or sports s/he is enrolled in. ***guided choice_85***

Q30. I provided my child with choices about the physical activity s/he does. ***guided choice_86***

Q31. I allowed my child to choose the physical activity/sports we do as a family (whether we go for a walk, hike, bike ride, or play an active game). ***guided choice_92***

Not relevant, child currently NOT active

Never, I prefer doing this myself

Rarely

Sometimes

Often

Most of the time

**SCORING**

Scoring for the short form by averaging the items for each construct as follow:

***CONTROL PHYSICAL ACTIVITY PARENTING PRACTICES DOMAIN***

- Coercive control (7 items)

***STRUCTURE PHYSICAL ACTIVITY PARENTING PRACTICES DOMAIN***

- Non directive support (3 items)
- Supportive expectation (3 items)
- Facilitation (3 items)
- Restrict inside physical activity (3 items)
- Allow unsupervised physical activity (3 items)

***AUTONOMY PHYSICAL ACTIVITY PARENTING PRACTICES DOMAIN***

- Autonomy support (2 items)
- Guided choice (4 items)
- Reward (3 items)

Inquiries about the instrument should be sent Dr. L.C. Mâsse at [lmasse@bcchr.ubc.ca](mailto:lmasse@bcchr.ubc.ca)
